# Construct validity of the PROMIS^®^ sexual function and satisfaction measures in patients with cancer

**DOI:** 10.1186/1477-7525-11-40

**Published:** 2013-03-11

**Authors:** Kathryn E Flynn, Bryce B Reeve, Li Lin, Jill M Cyranowski, Deborah Watkins Bruner, Kevin P Weinfurt

**Affiliations:** 1 Center for Patient Care and Outcomes Research (PCOR), Medical College of Wisconsin, Milwaukee, WI, USA; 2 Department of Psychiatry and Behavioral Sciences, Duke University Medical Center, Durham, NC, USA; 3 Department of Health Policy and Management, Gillings School of Global Public Health, University of North Carolina at Chapel Hill, Chapel Hill, NC, USA; 4 Duke Clinical Research Institute, Duke University School of Medicine, Durham, NC, USA; 5 Departments of Psychiatry and Psychology, University of Pittsburgh Medical Center, Pittsburgh, PA, USA; 6 Nell Hodgson Woodruff School of Nursing, Emory University, Atlanta, GA, USA

**Keywords:** Patient-reported outcome measures, Measurement, Validity, Quality of life, Male and female sexual dysfunction, Cancer

## Abstract

**Background:**

With data from a diverse sample of patients either in treatment for cancer or post-treatment for cancer, we examine inter-domain and cross-domain correlations among the core domains of the Patient-Reported Outcomes Measurement Information System Sexual Function and Satisfaction measures (PROMIS^®^ SexFS) and the corresponding domains from conceptually-similar measures of sexual function, the International Index of Erectile Function and the Female Sexual Function Index.

**Findings:**

Men (N=389) and women (N=430) were recruited from a tumor registry, oncology clinics, and an internet panel. The PROMIS SexFS, International Index of Erectile Function, and Female Sexual Function Index were used to collect participants’ self-reported sexual function. The domains shared among the measures include desire/interest in sexual activity, lubrication and vaginal discomfort/pain (women), erectile function (men), orgasm, and satisfaction. We examined correlations among different domains within the same instrument (discriminant validity) and correlations among similar domains measured by different instruments (convergent validity). Correlations demonstrating discriminant validity ranged from 0.38 to 0.73 for men and 0.48 to 0.74 for women, while correlations demonstrating convergent validity ranged from 0.62 to 0.83 for men and 0.71 to 0.92 for women. As expected, correlations demonstrating convergent validity were higher than correlations demonstrating discriminant validity, with one exception (orgasm for men).

**Conclusions:**

Construct validity was supported by convergent and discriminant validity in a diverse sample of patients with cancer. For patients with cancer who may or may not have sexual dysfunction, the PROMIS SexFS measures provide a comprehensive assessment of key domains of sexual function and satisfaction.

## Findings

### Background

The Patient-Reported Outcomes Measurement Information System^®^ Sexual Function and Satisfaction measures (PROMIS^®^ SexFS) are a newly-developed collection of measures to assess sexual function and satisfaction in men and women. They were developed based on extensive qualitative and quantitative research with cancer patients [1-4]. Recent reports support the measures’ content validity (based on patient and clinician focus group data regarding relevant sexual concepts) [3], face validity (based on cognitive interview data) [2], and discrimination between known groups of patients who had (or had not) asked a provider for help with sexual problems [4]. However, no published report has provided comprehensive evaluation of the convergent and discriminant validity of the core PROMIS SexFS measures.

The extent to which the PROMIS SexFS is used in research rests on the strength of evidence for the validity of the measures, that is, the degree to which the instrument measures what it is intended to measure [5-7]. In this paper we present evidence of construct validity in a diverse sample of patients with cancer. Specifically, we evaluate the relationship among the core PROMIS SexFS domains and subscales drawn from two frequently-used measures in cancer research, the International Index of Erectile Function [IIEF] [8] and the Female Sexual Function Index [FSFI] [9]). While a limited number of correlations between PROMIS SexFS and pre-specified IIEF and FSFI subscales were reported in a previous psychometric report [4], it did not provide comprehensive evidence of construct validity, including the magnitude of relationships across differing sexual function domains both within and across measures. In the current report we provide this evidence using a multi-trait multi-method approach [10]. We expect to observe stronger relationships between different measures of the same domain (e.g., sexual interest) as compared with relationships between different domains measured within the same instrument (e.g., sexual interest and orgasm).

## Methods

### Data collection

Data collection for the development of the PROMIS SexFS has been described in detail elsewhere [4]. Briefly, English-speaking adult patients who had been diagnosed with cancer were recruited from clinics, a tumor registry, and an internet panel. Participants completed static surveys online or by interviewer-administrated telephone survey. The institutional review board of the Duke University Medical Center approved this study, and all patient participants provided informed consent.

### Procedure

Most participants completed surveys online (79%), though participants were given the option to complete the survey by interviewer-administered telephone survey. Rates of survey completion were comparable by mode of administration. Those who opted for the telephone administration were significantly older (68 vs. 60 years), less likely to be married (64% vs. 81%), of lower household income level (<$50,000, 54% vs. 24%), and less likely to be white (71% vs. 89%). An evaluation of differential item functioning (DIF) by mode of administration suggested there was no DIF for phone vs. online administration [4].

### Measures

Items in the PROMIS SexFS, IIEF, and FSFI are rated on a 5-point scale regarding sexual function experienced over the past 30 days.

The PROMIS SexFS is a set of measures that includes separate domains for interest in sexual activity (4 items), global satisfaction with sex life (7 items), orgasm (1 item), erectile function (8 items, men only), lubrication (8 items, women only) and vaginal discomfort (10 items, women only). In the current version of the PROMIS SexFS measure, the 4 FSFI lubrication items are included in the PROMIS lubrication scale and the 3 FSFI pain items are included in the PROMIS vaginal discomfort scale. For items/domains describing function, respondents are treated as missing (not given a score) if they report no sexual activity in the past 30 days. More information about the measure and item wording is available on the Assessment Center (http://www.assessmentcenter.net).

The IIEF measures sexual dysfunction in men and includes subscales for sexual desire (2 items), intercourse satisfaction (3 items), overall satisfaction (2 items), orgasmic function (3 items), and erectile function (6 items). Respondents are given a score of zero on items/domains describing function if they report no sexual activity in the past 30 days.

The FSFI measures sexual dysfunction in women and includes subscales for sexual desire (2 items), arousal (4 items), satisfaction (3 items), orgasm (3 items), pain (3 items) and lubrication (4 items). Respondents are given a score of zero on items/domains describing function if they report no sexual activity in the past 30 days.

### Data analysis

Pearson’s product moment correlation coefficients were used to evaluate the strength of relationships across sexual function domains as part of a multi-trait multi-method test of convergent and discriminant validity [10]. Data were managed and analyzed using SAS version 9.2 (SAS Institute Inc, Cary, North Carolina).

## Results

**Figure 1:**
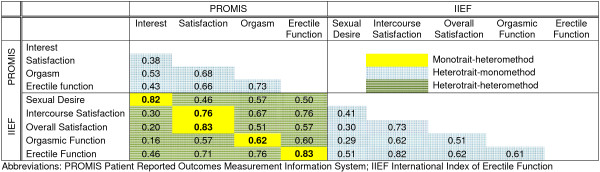
Correlations for men (N=390).

**Figure 2:**
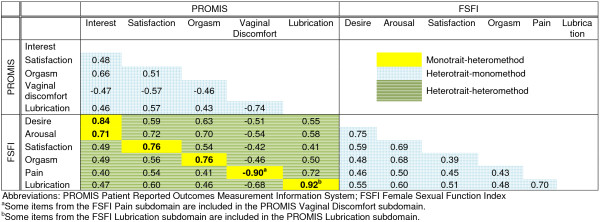
Correlations for women (N=429).

Sample characteristics are reported in Table 1. Of the full sample of 819 patients, 56% of men and 59% of women reported engaging in sexual activity over the past 30 days (this is not representative of rates of sexual activity in the broader US adult population). Figures 1 and 2 show correlations among domains for men and women, respectively. The correlations describing discriminant validity are shaded in blue gingham (within an instrument) or green stripes (from different instruments). The correlations describing convergent validity are shaded in solid yellow. All correlations were significantly different from zero (p<0.0001).

**Table 1:** Sample characteristics overall and by male/female sex (N=819)

**Characteristic**	**Total (N = 819)**	**Men (N = 389)**	**Women (N = 430)**
Age, mean ± SD, y	58.5 ± 11.8	61.4 ± 10.8	55.9 ± 12.2
Age group, No. (%)			
≤ 40 years	59 (7)	14 (4)	45 (10)
41 to 50 years	127 (16)	29 (7)	98 (23)
51 to 64 years	377 (46)	197 (51)	180 (42)
65 to 79 years	232 (28)	134 (35)	98 (23)
≥ 80 years	21 (3)	13 (3)	8 (2)
Race, No. (%)			
Black or African American	80 (10)	39 (10)	41 (10)
American Indian/Alaska Native	10 (1)	6 (2)	4 (<1)
Asian	12 (1)	2 (<1)	10 (2)
Native Hawaiian/Other Pacific Islander	2 (<1)	1 (<1)	1 (<1)
White	705 (87)	338 (87)	367 (86)
Multiple races or other	6 (<1)	1 (<1)	5 (<1)
Hispanic or Latino ethnicity, No. (%)	21 (3)	10 (3)	11 ( 3)
Educational attainment, No. (%)			
Less than high school	24 (3)	14 (4)	10 ( 2)
High school graduate/GED	100 (12)	35 (9)	65 (15)
Some college	255 (31)	122 (31)	133 (31)
College degree	229 (28)	114 (29)	115 (27)
Advanced degree (MA, PhD, MD)	211 (26)	104 (27)	107 (25)
Treatment status in past month, No. (%)			
None (ie, posttreatment follow-up)	526 (64)	290 (75)	236 (55)
Undergoing treatment	290 (36)	98 (25)	192 (45)
Radiation therapy	29 (4)	15 (4)	14 (3)
Hormonal therapy (eg, tamoxifen, anastrozole, leuprolide)	140 (17)	28 (7)	112 (26)
Chemotherapy (injection or oral)	116 (14)	46 (12)	70 (16)
Immunotherapy (eg, interferon)	9 (1)	2 (<1)	7 (2)
Other	36 (4)	17 (4)	19 (4)
Recurrence of cancer, No. (%)	151 (18)	68 (17)	83 (19)
Cancer spread to lymph nodes, No. (%)	202 (25)	68 (17)	134 (31)
Cancer spread to another area, No. (%)	134 (16)	57 (15)	77 (18)
Primary cancer diagnosis, No. (%)			
Breast cancer	252 (35)	1 (<1)	251 (62)
Colorectal	98(13)	57 (18)	41 (10)
Gynecologic cancer	29 (4)	–	29 (7)
Lung cancer	56 (8)	35 (11)	21 (5)
Prostate cancer	146 (20)	146 (45)	–
Other or Unknown	9 (1)	6 (2)	3 (<1)

Percentages may not sum to 100 because of rounding.

### Convergent validity – men

The PROMIS interest in sexual activity and IIEF sexual desire domains had large correlations (r=0.82), as did the PROMIS and IIEF erectile function domains (r=0.83) and the PROMIS and IIEF general satisfaction domains (r=0.83). The PROMIS orgasm and IIEF orgasmic function domains were less highly correlated (r=0.62).

### Discriminant validity – men

The PROMIS interest in sexual activity was the least highly correlated with the other PROMIS domains of erectile function, orgasm, and global satisfaction with sex life (r=0.38 to r=0.53). Erectile function was correlated with orgasm at r=0.73 and global satisfaction with sex life at r=0.66. Orgasm and global satisfaction with sex life were also correlated with each other at r=0.68.

The correlations across domains were smaller than the correlations within domains with the exception of the PROMIS orgasm domain, which had higher correlations with PROMIS Satisfaction (r=0.68) and with PROMIS Erectile Function (r=0.73) than with IIEF orgasm (r=0.62).

### Convergent validity – women

The PROMIS interest in sexual activity and FSFI desire domains had large correlations, as did the PROMIS and FSFI lubrication domains, and the PROMIS vaginal discomfort and FSFI pain domains (rs>0.8). Both the PROMIS and FSFI satisfaction domains and the PROMIS and FSFI orgasm domains were correlated at r=0.76.

### Discriminant validity – women

Among PROMIS domains, interest in sexual activity was moderately correlated with lubrication, vaginal discomfort, orgasm, and global satisfaction with sex life (r=0.46 to r=0.66). Lubrication was correlated with vaginal discomfort at r=−0.74, orgasm at r=0.43, and global satisfaction with sex life at r=0.57. Orgasm and global satisfaction with sex life were correlated with each other at r=0.51.

For women, all correlations within domains were higher than those across domains.

## Conclusions

Concurrent validity for the PROMIS SexFS measures was generally supported by convergent and discriminant validity in a diverse sample of patients with cancer. The correlations across different domains within the PROMIS measures were smaller than the correlations within similar domains across different sexual function measures. The exception to this was the PROMIS orgasm domain, which was more highly correlated with the satisfaction and erectile function domains than with the IIEF orgasm domain. Likewise, the IIEF orgasm domain was more highly correlated with the IIEF intercourse satisfaction and erectile function domains than with the PROMIS orgasm domain. The high correlations between orgasm and both satisfaction and erectile function should prompt consideration of the additional value of the orgasm domain for men, and the amount of data this domain provides beyond measures of erectile function and satisfaction. However, we note that focus group data support the inclusion of a separate orgasm domain for males, as men typically described orgasm as related to, but never conceptually the same as, erectile function or satisfaction. In the current version of the measure, the PROMIS orgasm domain includes a single item that asks respondents to rate their ability to have a satisfying orgasm. The IIEF includes one item on frequency of orgasm and one item on frequency of ejaculation. We wondered if there might be a higher correlation between the items asking about orgasm rather than ejaculation, but the correlation between the PROMIS and IIEF orgasm items was only slightly higher than the correlation between the PROMIS orgasm item and the IIEF ejaculation item (0.63 vs 0.58). These results suggest the need for broader assessment of the domain of orgasm, and we are testing additional items for version 2 of the PROMIS SexFS orgasm domain.

Our sample, while generally large and diverse, did not adequately represent people with cancers other than the most common ones (breast or prostate). People of non-white race and non-Hispanic ethnicity were also not well represented, nor were people with less than a college education.

In conclusion, scores on the PROMIS SexFS behaved as expected in relation to two well-regarded measures of sexual function, the IIEF and FSFI. This study adds to the validity data in support of the use of the freely-available PROMIS SexFS to comprehensively assess key domains of sexual function and satisfaction.

## Abbreviations

PROMIS^®^ SexFS: Patient-reported outcomes measurement information system^®^ sexual function and satisfaction measures; FSFI: Female sexual function index; IIEF: International index of erectile function.

## Competing interests

The authors declare that they have no competing interests.

## Authors’ contributions

KF contributed to the design of the study, interpretation of the results, and drafted the manuscript. BR, DB, and JC contributed to interpretation of the results and made critical revisions to the manuscript. LL contributed to the design of the study, performed the statistical analyses, and made critical revisions to the manuscript. KW contributed to the design of the study, the interpretation of the results, and made critical revisions to the manuscript. All authors read and approved the final manuscript.
